# Psychopathology in adults with copy number variants

**DOI:** 10.1017/S0033291721005201

**Published:** 2023-05

**Authors:** Rachael L. Adams, Alister Baird, Jacqueline Smith, Nigel Williams, Marianne B. M. van den Bree, David E. J. Linden, Michael J. Owen, Jeremy Hall, Stefanie C. Linden

**Affiliations:** 1 Division of Psychological Medicine and Clinical Neurosciences, Medical Research Council Centre for Neuropsychiatric Genetics and Genomics, Cardiff University, Cardiff, UK; 2 Department of Psychiatry and Neuropsychology, School for Mental Health and Neuroscience, Faculty of Health, Medicine and Live Sciences, Maastricht University, Maastricht, The Netherlands; 3 Department of Health, Ethics and Society, Care and Public Health Research Institute (CAPHRI), Faculty of Health, Medicine and Life Sciences, Maastricht University, Maastricht, The Netherlands

**Keywords:** Psychiatric genetics, anxiety disorders, mood disorders, psychosis, neurodevelopmental disorders, personality disorders, somatic phenotypes

## Abstract

**Background:**

Copy number variants (CNVs) have been associated with the risk of schizophrenia, autism and intellectual disability. However, little is known about their spectrum of psychopathology in adulthood.

**Methods:**

We investigated the psychiatric phenotypes of adult CNV carriers and compared probands, who were ascertained through clinical genetics services, with carriers who were not. One hundred twenty-four adult participants (age 18–76), each bearing one of 15 rare CNVs, were recruited through a variety of sources including clinical genetics services, charities for carriers of genetic variants, and online advertising. A battery of psychiatric assessments was used to determine psychopathology.

**Results:**

The frequencies of psychopathology were consistently higher for the CNV group compared to general population rates. We found particularly high rates of neurodevelopmental disorders (NDDs) (48%), mood disorders (42%), anxiety disorders (47%) and personality disorders (73%) as well as high rates of psychiatric multimorbidity (median number of diagnoses: 2 in non-probands, 3 in probands). NDDs [odds ratio (OR) = 4.67, 95% confidence interval (CI) 1.32–16.51; *p* = 0.017) and psychotic disorders (OR = 6.8, 95% CI 1.3–36.3; *p* = 0.025) occurred significantly more frequently in probands (*N* = 45; NDD: 39[87%]; psychosis: 8[18%]) than non-probands (*N* = 79; NDD: 20 [25%]; psychosis: 3[4%]). Participants also had somatic diagnoses pertaining to all organ systems, particularly conotruncal cardiac malformations (in individuals with 22q11.2 deletion syndrome specifically), musculoskeletal, immunological, and endocrine diseases.

**Conclusions:**

Adult CNV carriers had a markedly increased rate of anxiety and personality disorders not previously reported and high rates of psychiatric multimorbidity. Our findings support in-depth psychiatric and medical assessments of carriers of CNVs and the establishment of multidisciplinary clinical services.

## Introduction

Copy number variants (CNVs) are variations in the number of copies of chromosomal regions produced by microdeletions and microduplications due to meiotic misalignment. It has already been established that certain larger (>100 kilobase) CNVs confer increased risk for neurodevelopmental disorders (NDDs) such as autism spectrum disorder (ASD), schizophrenia (Scz), attention deficit hyperactivity disorder (ADHD) and intellectual disability (ID) (Coe, Girirajan, & Eichler, 2012; Kirov et al., 2014; Rees et al., 2014; Sebat, Levy, & McCarthy, 2009). Most research on the psychopathology associated with CNVs has been based on information from genome-wide association studies (Kirov et al., 2014; Rees et al., 2014) (with limited phenotyping information) or databases from clinical genetic services (Chawner et al., 2019) (with the concomitant focus on childhood clinical presentations). However, very few studies have conducted detailed phenotyping specifically in adults with CNVs.

There is evidence that the great majority of adult CNV carriers in the general population have not received a genetic diagnosis especially if they have not been affected by early-onset developmental disorders (Martin et al., 2020). Systematic population studies of adult CNV carriers have established cross-CNV association with cognitive impairment and depression (Kendall et al., 2017, 2019), but these reports are likely to have excluded many individuals with more severe outcomes. Consequently, there has been relatively little characterisation of the broader psychiatric phenotype across CNVs in adulthood. In the DEFINE study at Cardiff University, which recruited participants from 2014 to 2018, we conducted detailed clinical, cognitive and neural phenotyping (Dima et al., 2020; Drakesmith et al., 2019) of carriers of CNVs at loci that have been associated with Scz. The clinical phenotyping included detailed diagnostic interviews for axis 1 and axis 2 disorders and is described further in the Methods section. Based on the evidence of association from genome-wide studies we included deletions or duplications at 22q11.2, 15q11.2, 15q13.3, 16p11.2, 17q12, 1q21.1, 3q29, and 2p16.3 (Kirov et al., 2014). We also included deletions at 9q34 (Kleefstra syndrome) and 22q13.3 (Phelan McDermid syndrome), which are also associated with a range of NDDs. The DEFINE study thus offered an opportunity to investigate the relationship between CNVs and psychopathology and medical comorbidities in a deeply phenotyped adult sample. Here, we report on the psychiatric consequences of carrier status for pathogenic CNVs. In particular, we had the opportunity to assess psychopathology in a relatively high-functioning sample and ascertain the age of onset of mental disorder. This is the first study reporting on the prevalence and range of clinical syndromes in a deeply phenotyped cross-CNV cohort in adulthood. We addressed two principal questions: Is the psychiatric phenotype of neuropsychiatric CNVs in adulthood confined to the classically described NDDs or does it extend to anxiety and mood disorders? Do highly functioning CNV carriers (the majority of the non-proband group) also have high rates of psychopathology? Is their psychopathology attributable to the challenges of bringing up a child with a disabling genetic condition?

## Method

### Participant recruitment

The DEFINE study was set up in 2014 as an adult extension of the Experiences of Children with Copy Number Variants (ECHO) study at Cardiff University. Its aim is the psychopathological, clinical and biological characterisation of carriers of CNVs that confer risk for Scz. Because of the pleiotropy of these variants, assessments include the whole range of psychopathologies, detailed medical and developmental histories as well as a detailed cognitive assessment. The study was performed in accordance with the Declaration of Helsinki and approved by the Regional Ethics Committee of the National Health Service - approval: 14/WA/0035. It was promoted by charities for carriers of genetic variants and their families, social media and medical genetics services.

All participants provided written informed consent, or a personal consultee (who was always the main caregiver) provided consent on their behalf if they did not have the capacity to consent themselves. Subjects were included in the cohort if they met diagnostic criteria of either a confirmation of CNV via in-house Cardiff University genetic analysis, or confirmation evidence from medical genetics clinics which included breakpoints. We did not include participants who had more than one pathogenic CNV at the loci under investigation. Participants were asked to provide either a blood or saliva sample for genetic analysis, and if obtaining a sample was not possible, participants' medical genetics services were contacted for confirmation of the presence of a chromosomal disorder.

### Participant genotyping

To confirm CNV status, the National Centre for Mental Health (NCMH) at Cardiff University genotyped 101 participants (both probands (*n* = 39) – that is, the index cases for their families who originally came to the attention of medical genetics services - and non-probands (*n* = 62)) using the Illumina HumanCoreExome whole-genome SNP array that contains an additional 27 000 genetic variants at loci that had been previously implicated in neurological and psychiatric disease, which included CNVs. In the remainder of participants (*n* = 23; 6 probands, 17 non-probands), genotype was verified through medical genetics reports.

### Psychiatric and cognitive assessments

A battery of psychiatric assessments was administered to evaluate the presence of mental disorders. All diagnoses were confirmed by a consultant psychiatrist (SL) who either assessed the participants herself, or listened to the recordings of the interviews conducted by trained research psychologists. The data from a subset of participants with 1q21 deletion (*N* = 6) and duplication (*N* = 5) have been published separately as part of a multi-centre study (Linden et al., 2021). The psychiatric assessments took approximately 5 hours to complete for each participant and included the following battery: Psychiatric history taking and assessment of the present mental state; The Psychiatric Assessment Schedule for Adults with Developmental Disabilities Clinical Interview (PAS-ADD) (Moss et al., 1998), a well-established instrument to obtain all major psychiatric diagnoses under DSM IV criteria, designed to meet the particular challenges of assessment in people with ID but equally valid for use with the general population; The Structured Clinical Interview for DSM-IV AXIS II Personality Disorders (SCID-II); The Diagnostic Interview for Social and Communication Disorders (DISCO) (Wing, Leekam, Libby, Gould, & Larcombe, 2002), a semi-structured interview and accredited autism assessment tool, which was administered to confirm a diagnosis of autism if participant responses indicated the presence of autistic symptoms during the PAS-ADD interview; The DISCO could also be completed with a parent/ carer; The Structured Interview for Prodromal Symptoms (SIPS) (Miller et al., 2003), which assesses psychotic symptoms and identifies early symptoms or ‘prodromes’ of psychotic episodes.

The SIPS was only conducted in 53 participants (10 probands and 43 non-probands) because of intellectual limitations, time constraints or because a psychotic diagnosis had already been determined through the PAS-ADD. If the PAS-ADD indicated psychotic symptoms we also conducted the Scale for the Assessment of Positive Symptoms (SAPS) and the Scale for the Assessment of Negative Symptoms (SANS) (Andreasen, 1990). Because of the small number of patients with a psychotic diagnosis (*N* = 11) these results are not further reported. The Wechsler Abbreviated Scale of Intelligence (WASI) (Wechsler, 1999) was used to assess IQ. We also performed the Global Assessment of Functioning (GAF) as implemented in the SIPS, with two independent raters. We determined maternal education level as a proxy for socio-economic status (SES) (6 levels according to the British educational qualification system: 1: no formal qualifications; 2: GCSE, CSE; 3: A-levels, NVQ3; 4: Diploma, HND, NVQ4; 5: undergraduate degree; 6: postgraduate degree).

Participation in assessments was determined by the participant's personal circumstances, including capacity, availability of an informant and cognitive function. Occasionally, the severity of ID precluded obtaining meaningful information from assessments. Detailed participant histories and clinical interviews were used to establish the age at first psychiatric symptomology, or first psychiatric diagnosis. We recorded chronological timelines of psychiatric symptoms, including: first occurrence, continuity, and first treatment. In the non-proband group, which predominantly consisted of parents of clinically affected children, we established if their first episode of psychiatric symptoms preceded the birth of the index child, and if it preceded the genetic diagnosis of their child.

### Statistical analysis

Data were analysed in SPSS version 27 (IBM, Armonk/ NY, USA). We compared age and maternal education level between groups (probands and non-probands) with the Mann–Whitney-*U* test (variables were not normally distributed as determined with the Kolmogorov–Smirnov test). For the comparison of categorical variables (sex, special education) we used Pearson's χ^2^ test. We compared the rates of different diagnoses (any diagnosis, psychotic, neurodevelopmental, anxiety and mood disorder) and cognitive (IQ) and general (GAF) functional scores between proband and non-proband groups using generalised linear mixed models (GLMMs). We entered age, sex, IQ (except where IQ was the dependent variable) as fixed effects and family ID as a random effect. In addition, we computed the GLMMs for the sample after excluding all 22q11.2 deletion carriers.

## Results

### Participants

In total, 124 participants with a deletion (*N* = 83) or duplication (*N* = 41) were included in the analysis (see online Supplementary Table S1 for details of loci). At the time of recruitment, participants were between 18 and 76 years of age with a mean age of 36.5 years, s.d. = 13.247 years, 64% were female and 96% were Caucasian (3% were mixed race and 1% Asian). 45 participants (40 deletion and 5 duplication carriers; mean age = 27.8 (s.d. = 10.238), 48.9% female) were probands. The remaining participants were non-probands (*N* = 79, of whom 43 had a deletion and 36 had a duplication; mean age = 41.5 (s.d. = 12.170), 74.4% female). These were relatives of the probands (mostly parents but also siblings, grandparents, aunts, and uncles). Age (*U* = 616.5, *p* < 0.001) and gender (Pearson χ^2^ = 7.5, *p* = .006) were significantly different between groups and therefore entered as covariates in the regression model. The average time spent in mainstream education was similar for probands (13.5 years) and non-probands (13 years), but the proband group had a much higher proportion of participants who had received special education (36 [80%] had been in special education for more than 1 year, compared to 15 [19%] in the non-proband group) (Pearson χ^2^ = 44.1, *p* < 0.001). Groups did not differ on maternal education score (mean/median for probands: 2.9/2; mean/median for non-probands: 2.7/2; *U* = 948.0; *p* = 0.58). We had 95 families in total, thus most of them (*N* = 76) contributed only one case. Many families contributed no probands but only one non-proband because we partly recruited parents of index cases, where the index case was a minor and thus not included in this study.

### Psychopathology data

This cohort had a very high rate of psychopathology, with 85% of participants having a psychiatric diagnosis (Table 1). Amongst the axis 1 disorders, mood disorder (42%), anxiety (47%) and NDDs (48%) were most frequent. Rates of personality disorder were even higher (73%). The most frequent personality disorders were Avoidant (42%) and Depressive (19%) Personality Disorder, followed by Paranoid (12%), Obsessive Compulsive (12%), Borderline (10%), Schizotypal (10%), Passive Aggressive (10%), Anti-Social (8%) and Schizoid (6%) Personality Disorder (patients could be diagnosed with more than one personality disorder). We also split the data into probands and non-probands and provided separate columns for the probands and non-probands excluding all 22q DS carriers (which was the largest group of carriers of an individual CNV and contributed particularly to the diagnoses of psychosis). Table 1 also shows the general population rates for the different psychiatric diagnoses. In the online Supplementary Material, we provide a separate table with psychiatric diagnoses for each of the included CNVs, deletions and duplications (online Supplementary Table S2).

**Table 1.** Psychiatric diagnoses according to DSM-IV, in the whole sample, probands, non-probands, and probands and non-probands without all 22q DS carriers

Psychiatric diagnoses (DSM-IV)	Number diagnosed [%], *N* = 124	Number of probands diagnosed [%], *N* = 45	Number of non-probands diagnosed [%], *N* = 79	Number of probands diagnosed [%], 22q DS excluded, *N* = 25	Number of non-probands diagnosed [%], 22q DS excluded, *N* = 66	Population rates from literature (Ranges are 95% confidence intervals)
Any psychiatric diagnosis	106 [85]	41 [91]	65 [82]	23 [92]	55 [83]	Male: 28.6-34.8%; Female: 29.0-35.3% (Steel et al., 2014)
Any anxiety disorder	58 [47]	16 [36]	42 [53]	8 [32]	34 [52]	Male: 8.8-11.6%; Female: 16.2-20.4% (Steel et al., 2014)
Any mood disorder	52 [42]	15 [33]	37 [47]	8 [32]	31 [47]	Male: 6.3-8.5%; Female: 12.4-15.9% (Steel et al., 2014)
Any NDD^a^	59 [48]	39 [87]	20 [25]	22 [88]	18 [27]	Boys: 18%; Girls: 9.5% (Boyle et al., 2011)
Any psychotic disorder	11 [9]	8 [18]	3 [4]	2 [8]	3 [5]	0.56–0.62% (Moreno-Küstner et al., 2018)
Schizophrenia	8 [7]	7 [16]	1 [1]	2 [8]	1 [2]	
Schizoaffective disorder	2 [2]	1 [2]	1 [1]	0	1 [2]	
Other psychotic disorder	1 [1]	0	1 [1]	0	1 [2]	
Any personality disorder^b^	38 [73]	5 [83]	33 [72]	1 [100]	30 [83]	8.01-7.02% (Volkert et al., 2018)
Substance use disorder	7 [6]	2 [4]	5 [6]	2 [8]	5 [8]	Males: 2.8–3.3%; Females: 1.3–1.5% (GBD 2017 Disease and Injury Incidence and Prevalence Collaborators, 2018)
Conduct disorder	3 [2]	0	3 [4]	0	3 [5]	1.6–2.9% (Polanczyk, Salum, Sugaya, Caye, & Rohde, 2015)
Any eating disorder	6 [5]	1 [2]	5 [6]	0	5 [8]	Males: 0.8-6.5%; Females: 3.3-18.6% (Galmiche, Déchelotte, Lambert, & Tavolacci, 2019)

a Neurodevelopmental disorders, including autism, ADHD and intellectual disability but not schizophrenia.

b *N* = 52 (SCID-II only conducted with 52 individuals, of whom six (of whom five had 22q DS) were probands, 46 (of whom 10 had 22q DS) were non-probands.

Rates of psychopathology did not differ significantly (OR = 0.4, *p* = 0.31) between probands (91%) and non-probands (82%), but the distribution of diagnoses was different. Probands had significantly higher rates of neurodevelopmental disorder (OR = 4.66, *p* = 0.019) and psychosis (OR = 15.30, *p* = 0.01) whereas non-probands had particularly high rates of mood (47%) and anxiety disorder (53%), although the difference between the two carrier groups was not significant (anxiety: OR = 0.49, *p* = 0.24; mood disorder: OR = 1.46, *p* = 0.60). We also found significant negative associations between full-scale IQ (FSIQ) and any diagnosis (OR = 0.96, *p* = 0.047) and any neurodevelopmental diagnosis (OR = 0.96, *p* = 0.025), a significant increase with age for any psychotic disorder (OR = 1.07, *p* = 0.015) and a significant sex effect (higher in females) for anxiety (OR = 2.64, *p* = 0.035) and mood disorders (OR = 3.66, *p* = 0.009). For more details on the results of the GLMM analysis see online Supplementary Table S3. When removing the 33 participants with 22q11, the effects for NDD and psychosis continued to point in the same direction (higher for probands), but lost statistical significance (see online Supplementary Table S4).

**Fig. 1.**
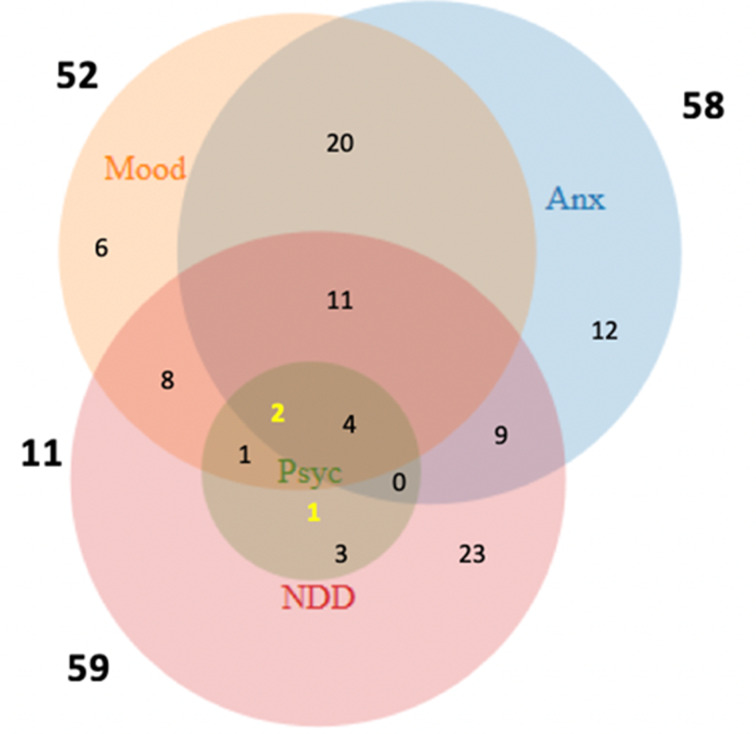
Venn diagram, demonstrating comorbidity between the main groups of diagnoses (mood disorder (Mood): *N* = 52; anxiety disorder (Anx): *N* = 58; neurodevelopmental disorder (NDD): *N* = 59; psychosis (Psyc): *N* = 11). Three participants (highlighted yellow) had a diagnosis of psychosis but no neurodevelopmental disorder.

There was considerable co- and multimorbidity, with participants having up to 11 (non-proband; median: two) or eight (proband; median: three) diagnoses. The overlap of the four main diagnostic categories (mood, anxiety, psychotic, NDD) is illustrated in Fig. 1. For the non-probands who were parents of probands (*N* = 51), the onset of psychopathology always predated the genetic diagnosis of the index child, and for all except five it pre-dated the birth of the index child.

### Prodromal syndromes

Of the 53 participants tested with the SIPS, 17 (2 probands out of 10 tested; 15 non-probands out of 43 tested) had a prodromal syndrome (15: Attenuated Positive Symptom Psychosis-Risk Syndrome; 3: Brief Intermittent Psychotic Symptom Psychosis-Risk Syndrome; one participant had both). Three of them were carriers of the 22q11.2 deletion. The difference in frequency of a prodromal syndrome between groups (probands *v.* non-probands) was not significant (Fisher exact test statistic 0.4711). All individuals meeting the criteria for a prodromal syndrome were experiencing symptoms at the time of assessment (for APS, these symptoms had either begun within the past year, or increased in severity in the past 12 months) (McGlashan, Walsh, & Woods, 2010).

### Developmental data

Rates of prematurity were similar to those in the general population (around 10% (Purisch & Gyamfi-Bannerman, 2017)). However, there were high rates of early feeding problems (49/124 [40%]), speech and language delay (54/ 124 [44%]) and motor delay (33/124 [27%]), particularly in the proband group. 10/124 participants (22% of all probands) fulfilled criteria for Developmental Coordination Disorder. For further details see Table 2.

**Table 2.** Neurodevelopmental data for the whole sample, including weight at birth, length of pregnancy, early feeding problems, speech and language delay, motor delay, and developmental coordination disorder

	Weight (kg)			Length of pregnancy (wks)			Early Feeding problems	Speech and Language delay	Motor delay	Developmental coordination disorder
	Underweight <2.5	Normal 2.5-4.5	Overweight >4.5	Premature <37	Normal ⩾37	Unknown				
All *N* = 124 [%]	16 [13]	107 [86]	1 [1]	12 [10]	82 [66]	30 [24]	49 [40]	54 [44]	33 [27]	10 [8]
Female *N* = 80 [%]	12 [15]	67 [84]	1 [1]	9 [11]	54 [68]	17 [21]	26 [33]	30 [38]	17 [21]	3 [4]
Male *N* = 44 [%]	4 [9]	40 [91]	.	3 [7]	28 [64]	13 [30]	23 [52]	24 [55]	16 [36]	5 [11]
Proband *N* = 45 [%]	8 [18]	36 [80]	1 [2]	1 [2]	32 [71]	12 [27]	32 [71]	40 [89]	26 [58]	10 [22]
Non-proband *N* = 79 [%]	8 [10]	71 [90]	.	11 [14]	50 [63]	18 [23]	17 [22]	14 [18]	7 [9]	.
22q11.2 del *N* = 33 [%]	7 [21]	26 79]	.	4 [12]	20 [61]	9 [27]	17 [52]	21 [64]	11 [33]	1 [1]

### IQ data

Mean FSIQ, verbal (VIQ) and performance IQ (PIQ) of probands were in the Borderline range for probands and in the average range for non-probands (see Table 3, all group differences significant at p(2-tailed)<0.001).

**Table 3.** Mean (standard deviations) of IQ values for probands and non-probands

	Probands, *N* = 32^a^	Non-probands, *N* = 76^a^	*U*-statistic
VIQ	72.9 (18.199)	91.9 (16.444)	544.000 (*p* < 0.001)
PIQ	78.1 (16.476)	99.8 (14.559)	397.500 (*p* < 0.001)
FSIQ	73.5 (16.881)	95.4 (15.594)	422.000 (*p* < 0.001)

a 16 participants from the overall sample did not complete cognitive assessments because of severe intellectual disability (*N* = 11, all of them probands) or other reasons, for example time pressure on assessment days (*N* = 5).

### GAF scores

GAF scores were available for 42 probands (mean = 40.25, s.d. = 20.988) and 75 non-probands (mean = 65.85, s.d. = 16.270). The group difference was significant (*t* = 5.62; *p* < 0.001).

### Somatic diagnoses

We found a high rate of somatic diagnoses, particularly from the categories of musculoskeletal syndromes (52.4% of participants had at least one diagnosis), immunological problems (51.6% had recurrent infections), cardiovascular disorders (32.3%), endocrine (27.4%) and respiratory disorders (25.8%). Some of the syndromal findings were prominent in the 22q11.2 subgroup, particularly congenital heart malformations and cleft palate, but carriers of the other CNVs also had high rates of somatic comorbidities, for example in the areas of endocrine and musculoskeletal diseases and epilepsy (online Supplementary Table S5).

## Discussion

### High rates of psychopathology in adult CNV carriers

We found overall high rates of psychopathology in this group of adult CNV carriers, both in probands, defined as the index cases originally referred to medical genetics, and in their relatives who also carried the CNV, who formed the non-proband group. This is remarkable because the non-probands were not clinically ascertained. Their rate of psychiatric diagnoses (lifetime prevalence) was still over two times higher than the highest estimates for the general population in European high-income countries (95% confidence interval: 30.7–39.9%) (Steel et al., 2014). In the non-probands, we found particularly high prevalence for both anxiety and mood disorders, with rates greater than double the upper boundary of general population estimates, even after adjustment for the disproportionately higher number of female relatives of probands in our sample; 53% for anxiety disorders in the non-probands compared with 16.2–20.4%, and 47% for mood disorder compared with 12.4–15.9% in the general population (Steel et al., 2014). Associations have been found between genetic risk for Scz (polygenic score) and anxiety traits (Jones et al., 2016), which may suggest some overlap in the clinical and subclinical phenotypes (and potentially the mechanisms) across rare and common risk variants. However, the clinical phenotype in the non-probands was not confined to anxiety and mood disorders but also included high rates of autism (10%) and prodromal symptoms (35% of tested participants, see below for further discussion).

These results are relevant for the further development of psychiatric genetics, both in terms of research and service development: Non-probands are people whose genotype status would not have been detected, had it not been for the more severe phenotype (and ensuing genetic diagnosis) of their child. This might not be a problem for them if they are clinically not affected at all – but we found that many of them still carry a high load of psychopathology. This is interesting for gene-phenotype research, particularly into the sources of variable penetrance and the role of genetic modifiers. Yet it can also inform service development, for example in relation to the question whether parents of children diagnosed with a CNV disorder who are carriers of the CNV themselves should be offered clinical support. As expected, given the clinical ascertainment of probands, rates of autism and other NDDs (including ID) were much higher in this group, with 87% diagnosed with at least one NDD. There was also a much higher rate of a psychotic disorder (18%) than expected for the general population (generally estimated at below 1% (Moreno-Küstner, Martín, & Pastor, 2018)) although this was largely driven by the 22q11.2 deletion subgroup (7 out of the 11 participants with psychotic disorders were 22q11.2 del carriers; 22.2% of 22q11.2 del carriers had a psychotic disorder, *v.* 4.4% in the group of other CNVs). The much higher rates for ID than psychosis in the overall cohort match the reported higher penetrance for ID/developmental delay for the included CNVs (Kirov et al., 2014).

We found high rates of prodromal syndromes of Scz particularly in the non-proband group (see online Supplementary Fig.). This is remarkable because this syndrome is rare in the general population [e.g. 0.3% in a recent survey of Chinese college students (Wu et al., 2021)]. Although high rates of prodromal symptoms have been reported previously in carriers of the 22q11.2 deletion (Tang et al., 2014; Weisman et al., 2017) our results are novel in indicating similarly high rates in a non-clinically ascertained sample, and also across a wider spectrum of CNVs. We did not have longitudinal data to assess rates of conversion to fully-fledged psychosis but would suggest that this question should be addressed by future research, and also monitored by clinical services for people with pathogenic CNVs.

There is almost no information about personality disorders in copy number variant carriers in the published literature. The high rate of personality disorders in our sample of 73%, over four times the upper limit of the general population estimates for any personality disorder (95% CI, 8.01–17.02%) (Volkert, Gablonski, & Rabung, 2018), is a novel finding. 50% had at least one cluster C personality disorder, which is approximately ten times the population rate (Winsper et al., 2020). These unexpectedly high rates of personality disorder may be linked to the heightened vulnerability during the crucial brain maturation period that is generally associated with CNV syndromes. These findings open up new perspectives for research (more focus on axis 2 assessments) and clinical services (consideration of genetic testing for patients with a personality disorder).

The care for a child with functional impairments can be stressful and result in an increased burden of mental illness (Chambers & Chambers, 2015), including anxiety and mood disorders (which were particularly prominent in our group of non-probands/ relatives). It is therefore important to point out that we largely excluded effects of reverse causation in the psychopathology of the non-proband group, which included a high proportion of parents of clinically affected children, by ascertaining that the onset of psychopathology preceded the diagnosis, and in most cases also the birth of their child. Furthermore, most of the non-probands only discovered that they had a CNV after a relative's early-onset developmental disorder and diagnosis of a CNV prompted a referral to clinical genetics to assess heritability.

### Somatic phenotypes

We confirmed the known features of the somatic syndrome of 22q11.2 deletion (e.g. cardiac malformations, cleft palate, hypothyroidism, scoliosis, recurrent infections) (Fung et al., 2015; McDonald-McGinn et al., 2015). We also found high rates of congenital malformations and other somatic comorbidities in the carriers of the other CNVs although numbers for individual CNVs were too small to determine any specific association. Particularly salient and common features were endocrine, immunological and musculoskeletal abnormalities. We also found high rates of epilepsy in the non-22q11.2 deletion group (24%), which was even higher than that reported in a young sample with 22q11.2 deletion syndrome (Eaton et al., 2019).

Because of the size of our cohort, we could not ascertain any specific diagnostic patterns associated with particular CNVs (apart from 22q11.2 deletion), which limits the possibilities of mechanistic inferences. However, the convergence of such a wide range of CNVs (with widely varying numbers of genes implicated – from single gene variants (2p16.3: *neurexin*; 22q13.3: *SHANK3*) to variants involving almost 100 genes (the 3Mb variant of 22q11.2) on common psychiatric and somatic phenotypes is in line with a previous study in children (Chawner et al., 2021b) and opens up worthwhile terrain for further investigation of the downstream mechanisms of these genetic variants.

### Limitations

A limitation of this work is the cross-CNV nature of the cohort which does not allow us to answer the question whether individual loci are associated with specific psychopathology. Another potential limitation is the lack of a non-CNV control group but we would argue that estimates obtained from large epidemiological surveys (as referenced above) are a more accurate reflection of population base rates. Finally, the sample size was limited because this was a newly set-up adult cohort and, given the tradition of the research group, had a strong representation of 22q11.2 deletion carriers. When removing these from the analysis the diagnostic differences between probands and non-probands became non-significant. Thus, expansion through international collaboration will be needed to obtain more robust estimates of rates of psychopathology and in order to disentangle potential CNV-specific phenotypes. Another limitation of the study was that prodromal syndromes and personality disorders (and IQ) could not be assessed in some participants with ID.

## Conclusion

The main finding from this paper was the high rate of disorders that may not present in childhood but only emerge during adolescence and adulthood, which highlights the need for a lifetime perspective on CNVs. Even CNV carriers with normal IQ and a high level of functioning have a high rate of mental and personality disorders (and often also somatic co-morbidities), which pose particular challenges to management (Chawner, Watson, & Owen, 2021a). Knowing about CNV status can be important for both patients and healthcare professionals because it provides an explanation for complex clinical needs and helps with streamlining support. Our data thus support the further expansion of genetic testing in patients with complex psychopathologies (Thygesen et al., 2018). A clinical consequence of our findings could be the increased collaboration between medical genetics and mental health services, for example through the establishment of mental health screening and specialised services for genetically affected parents of probands with CNVs.

## Supplementary material

For supplementary material accompanying this paper visit https://doi.org/10.1017/S0033291721005201. click here to view supplementary material
