# Supplementary material for: Psychopathology in adults with copy number variants
Source: Psychol Med. 2022 Feb 11;53(7):3142–9. doi: 10.1017/S0033291721005201 (PMC10244007; doi:10.1017/S0033291721005201)
Supplement: Supplementary file 1 [file S0033291721005201sup001.zip › S0033291721005201sup001.docx]

Supplementary Figure

Flowchart of diagnosis of prodromal syndromes with the Structured Interview of Psychosis-risk Syndromes (SIPS). BLIPS: Brief Limited Intermittent Psychotic Symptoms; APS: Attenuated Positive Symptoms; GRDPS: Genetic Risk and Deterioration Prodromal Syndrome.

*one participant had a research diagnosis of BLIPS and APS
