# Supplementary material for: Psychopathology in adults with copy number variants
Source: Psychol Med. 2022 Feb 11;53(7):3142–9. doi: 10.1017/S0033291721005201 (PMC10244007; doi:10.1017/S0033291721005201)
Supplement: Supplementary file 1 [file S0033291721005201sup001.zip › S0033291721005201sup002.docx]

Table S1: Distribution of CNVs

| Copy Number Variant | Cases with CNV  N=124 | Probands  N=45 | Non-probands  N=79 |
| --- | --- | --- | --- |
| 1q21.1 Deletion | 9 | 2 | 7 |
| 1q21.1 Duplication | 13 | 2 | 11 |
| 2p16.3 Deletion | 3 | 2 | 1 |
| 3q29 Deletion | 3 | 3 | - |
| 9q34.3 Deletion | 5 | 5 | - |
| 15q11.2 Deletion | 14 | 3 | 11 |
| 15q11.2 Duplication | 9 | 2 | 7 |
| 15q13.3 Deletion | 8 | 3 | 5 |
| 15q13.3 Duplication | 8 | - | 8 |
| 16p11.2 Deletion | 7 | 1 | 6 |
| 16p11.2 Duplication | 1 | - | 1 |
| 17q12 Duplication | 6 | 1 | 5 |
| 22q11.2 Deletion | 33 | 20 | 13 |
| 22q11.2 Duplication | 4 | - | 4 |
| 22q13.3 Deletion | 1 | 1 | - |
