# Supplementary material for: Psychopathology in adults with copy number variants
Source: Psychol Med. 2022 Feb 11;53(7):3142–9. doi: 10.1017/S0033291721005201 (PMC10244007; doi:10.1017/S0033291721005201)
Supplement: Supplementary file 1 [file S0033291721005201sup001.zip › S0033291721005201sup003.docx]

|  | | | | | **Number of CNV carriers diagnosed (%)** | | | | | | | | | | | | | | |
| --- | --- | --- | --- | --- | --- | --- | --- | --- | --- | --- | --- | --- | --- | --- | --- | --- | --- | --- | --- |
| **Psychiatric Diagnosis DSM IV** | Number assessed | Number diagnosed (%) | Number of probands diagnosed (%)  n=45 | Number of non-probands diagnosed (%)  n=79 | 22q11.2 del  n=33 | 22q11.2 dup  n=4 | 22q13.3 del  n=1 | 1q21.1 del  n= 9 | 1q21.1  dup  n= 13 | 2p16.3  del  n=3 | 3q29 del  n=3 | 9q34.3 n=5 | 15q11.2 del  n=14 | 15q11.2q12 dup  n=9 | 15q13.1 del  n=8 | 15q13.3 dup  n=8 | 16p11.2 del  n=7 | 16p11.2  dup  n=1 | 17q12  dup  n=6 |
| **Psychiatric Diagnosis**  **(Any)** | 124 | 106 (85) | 41 (91) | 65 (82) | 28 (85) | 2 (50) | 1 (100) | 7 (78) | 11 (85) | 3 (100) | 3 (100) | 5 (100) | 12 (86) | 8 (89) | 7 (86) | 6 (75) | 6 (86) | 1 (100) | 6 (100) |
| **Anxiety Disorder**  **(Any)** | 124 | 58 (47) | 16 (36) | 42 (53) | 16 (49) | - | - | 3 (33) | 9 (69) | 1 (33) | 1 (33) | 2 (40) | 8 (57) | 6 (67) | 3 (38) | 5 (63) | - | 1 (100) | 3 (50) |
| **Mood**  **Disorder**  **(Any)** | 124 | 52 (42) | 15 (33) | 37 (47) | 13 (39) | 1 (25) | 1 (100) | 5 (56) | 9 (69) | - | 1 (33) | 2 (40) | 4 (29) | 3 (33) | 3 (38) | 5 (63) | 1 (14) | - | 4 (67) |
| **Behavioural Disturbance (Any)** | 124 | 9 (7) | 2 (4) | 7 (9) | - | - | - | 2 (22) | 3 (23) | - | - | - | 1 (7) | - | - | 1 (13) | 1 (14) | - | 1 (17) |
| **Eating**  **Disorder**  **(Any)** | 124 | 6 (5) | 1 (2) | 5 (6) | 1 (3) | 1 (25) | - | - | 2 (15) | - | - | - | - | - | - | 1 (13) | 1 (14) | - | - |
| **Personality Disorder ***  **(Any)** | 67 | 38 (57) | 5 (71) *7* | 33 (55) *60* | 7 (47) *15* | - | - | 3 (50) *6* | 9 (82) *11* | - | - | - | 6 (86) *7* | 2 (33) *6* | 1 (33) *3* | 5 (71) *7* | 2 (40) *5* | 1 (100) | 2 (67) *3* |
| **Psychotic Disorder**  **(Any)** | 124 | 11 (9) | 8 (18) | 3 (4) | 6 (18) | 1 (25) | - | - | 2 (15) | - | - | 1 (20) | - | - | - | - | - | - | 1 (17) |
| **Schizophrenia** | 124 | 8 (7) | 7 (16) | 1 (1) | 5 (15) | - | - | - | 1 (8) | - | - | 1 (20) | - | - | - | - | - | - | 1 (17) |
| **Schizoaffective Disorder** | 124 | 2 (2) | 1 (2) | 1 (1) | 1 (3) | - | - | - | 1 (8) | - | - | - | - | - | - | - | - | - | - |
| **Other Psychotic Disorder Diagnosis** | 124 | 1 (1) | 0 | 1 (1) | - | 1 (25) | - | - | - | - | - | - | - | - | - | - | - | - | - |
| **Neurodevelop-mental Disorder**  **(Any)** | 124 | 59 (48) | 39 (87) | 20 (25) | 19 (58) | 1 (25) | 1 (100) | 2 (22) | 4 (31) | 3 (100) | 3 (100) | 5 (100) | 6 (43) | 5 (56) | 4 (50) | 2 (25) | 2 (29) | - | 2 (33) |
| **Intellectual Disability** | 124 | 32 (26) | 29 (64) | 3 (4) | 13 (39) | - | 1 (100) | 1 (11) | - | 2 (67) | 1 (33) | 5 (100) | 3 (21) | - | 3 (38) | - | 2 (29) | - | 1 (17) |
| **Autism Spectrum Disorder** | 124 | 31 (25) | 23 (51) | 8 (10) | 9 (27) | - | 1 (100) | 1 (11) | 3 (23) | 1 (33) | 3 (100) | 3 (60) | 2 (14) | 3 (33) | 1 (13) | 1 (13) | 1 (14) | - | 2 (33) |

* In italics the number of individuals assessed with the SCID-II

Table S2: Rates of psychiatric diagnoses in CNV carriers; Overall, probands, non-probands, and individual CNVs, deletions and duplications
