# Supplementary material for: Psychopathology in adults with copy number variants
Source: Psychol Med. 2022 Feb 11;53(7):3142–9. doi: 10.1017/S0033291721005201 (PMC10244007; doi:10.1017/S0033291721005201)
Supplement: Supplementary file 1 [file S0033291721005201sup001.zip › S0033291721005201sup004.docx]

**Table S3: GLMM model parameters (psychopathology)**

Any psychiatric diagnosis

| **Fixed Coefficients^a^** | | | | | | | | | |
| --- | --- | --- | --- | --- | --- | --- | --- | --- | --- |
| Model Term | Coefficient | Std. Error | t | Sig. | 95% Confidence Interval | | Exp(Coefficient) | 95% Confidence Interval for Exp(Coefficient) | |
|  |  |  |  |  | Lower | Upper |  | Lower | Upper |
| Intercept | 7.090 | 2.4579 | 2.884 | .005 | 2.215 | 11.965 | 1199.773 | 9.163 | 157097.631 |
| prob=1 | -.916 | .9027 | -1.015 | .312 | -2.707 | .874 | .400 | .067 | 2.396 |
| prob=0 | 0^b^ | . | . | . | . | . | . | . | . |
| Gender=Male | -.058 | .6195 | -.093 | .926 | -1.286 | 1.171 | .944 | .276 | 3.225 |
| Gender=Female | 0^b^ | . | . | . | . | . | . | . | . |
| age | -.032 | .0248 | -1.279 | .204 | -.081 | .017 | .969 | .922 | 1.018 |
| FSIQ | -.042 | .0207 | -2.014 | .047 | -.083 | -.001 | .959 | .920 | .999 |
| Probability distribution: Binomial  Link function: Logit^a^ | | | | | | | | | |
| a. Target: anypsy | | | | | | | | | |
| b. This coefficient is set to zero because it is redundant. | | | | | | | | | |

Any psychotic disorder

| **Fixed Coefficients^a^** | | | | | | | | | |
| --- | --- | --- | --- | --- | --- | --- | --- | --- | --- |
| Model Term | Coefficient | Std. Error | t | Sig. | 95% Confidence Interval | | Exp(Coefficient) | 95% Confidence Interval for Exp(Coefficient) | |
|  |  |  |  |  | Lower | Upper |  | Lower | Upper |
| Intercept | -5.270 | 2.4820 | -2.123 | .036 | -10.192 | -.347 | .005 | 3.747E-5 | .707 |
| prob=1 | 2.728 | 1.0351 | 2.636 | .010 | .675 | 4.781 | 15.304 | 1.965 | 119.215 |
| prob=0 | 0^b^ | . | . | . | . | . | . | . | . |
| Gender=Male | -.008 | .6895 | -.011 | .991 | -1.375 | 1.360 | .992 | .253 | 3.895 |
| Gender=Female | 0^b^ | . | . | . | . | . | . | . | . |
| age | .071 | .0286 | 2.467 | .015 | .014 | .127 | 1.073 | 1.014 | 1.136 |
| FSIQ | -.008 | .0208 | -.391 | .697 | -.049 | .033 | .992 | .952 | 1.034 |
| Probability distribution: Binomial  Link function: Logit^a^ | | | | | | | | | |
| a. Target: anypsychot | | | | | | | | | |
| b. This coefficient is set to zero because it is redundant. | | | | | | | | | |

Any neurodevelopmental disorder

| **Fixed Coefficients^a^** | | | | | | | | | |
| --- | --- | --- | --- | --- | --- | --- | --- | --- | --- |
| Model Term | Coefficient | Std. Error | t | Sig. | 95% Confidence Interval | | Exp(Coefficient) | 95% Confidence Interval for Exp(Coefficient) | |
|  |  |  |  |  | Lower | Upper |  | Lower | Upper |
| Intercept | 2.863 | 1.6674 | 1.717 | .089 | -.444 | 6.170 | 17.508 | .641 | 477.964 |
| prob=1 | 1.539 | .6437 | 2.391 | .019 | .262 | 2.816 | 4.661 | 1.300 | 16.707 |
| prob=0 | 0^b^ | . | . | . | . | . | . | . | . |
| Gender=Male | .972 | .5108 | 1.904 | .060 | -.041 | 1.986 | 2.645 | .960 | 7.283 |
| Gender=Female | 0^b^ | . | . | . | . | . | . | . | . |
| age | -.023 | .0218 | -1.068 | .288 | -.066 | .020 | .977 | .936 | 1.020 |
| FSIQ | -.035 | .0155 | -2.276 | .025 | -.066 | -.005 | .965 | .936 | .995 |
| Probability distribution: Binomial  Link function: Logit^a^ | | | | | | | | | |
| a. Target: anyneurodev | | | | | | | | | |
| b. This coefficient is set to zero because it is redundant. | | | | | | | | | |

Any anxiety disorder

| **Fixed Coefficients^a^** | | | | | | | | | |
| --- | --- | --- | --- | --- | --- | --- | --- | --- | --- |
| Model Term | Coefficient | Std. Error | t | Sig. | 95% Confidence Interval | | Exp(Coefficient) | 95% Confidence Interval for Exp(Coefficient) | |
|  |  |  |  |  | Lower | Upper |  | Lower | Upper |
| Intercept | 1.604 | 1.4692 | 1.091 | .278 | -1.310 | 4.517 | 4.971 | .270 | 91.595 |
| prob=1 | -.723 | .6074 | -1.191 | .237 | -1.928 | .481 | .485 | .145 | 1.618 |
| prob=0 | 0^b^ | . | . | . | . | . | . | . | . |
| Gender=Male | -.969 | .4541 | -2.134 | .035 | -1.870 | -.068 | .379 | .154 | .934 |
| Gender=Female | 0^b^ | . | . | . | . | . | . | . | . |
| age | -.030 | .0189 | -1.566 | .120 | -.067 | .008 | .971 | .935 | 1.008 |
| FSIQ | 6.965E-5 | .0134 | .005 | .996 | -.027 | .027 | 1.000 | .974 | 1.027 |
| Probability distribution: Binomial  Link function: Logit^a^ | | | | | | | | | |
| a. Target: anyanx | | | | | | | | | |
| b. This coefficient is set to zero because it is redundant. | | | | | | | | | |

Any mood disorder

| **Fixed Coefficients^a^** | | | | | | | | | |
| --- | --- | --- | --- | --- | --- | --- | --- | --- | --- |
| Model Term | Coefficient | Std. Error | t | Sig. | 95% Confidence Interval | | Exp(Coefficient) | 95% Confidence Interval for Exp(Coefficient) | |
|  |  |  |  |  | Lower | Upper |  | Lower | Upper |
| Intercept | -1.883 | 1.5292 | -1.231 | .221 | -4.916 | 1.150 | .152 | .007 | 3.158 |
| prob=1 | .381 | .6282 | .607 | .545 | -.865 | 1.627 | 1.464 | .421 | 5.089 |
| prob=0 | 0^b^ | . | . | . | . | . | . | . | . |
| Gender=Male | -1.297 | .4860 | -2.669 | .009 | -2.261 | -.333 | .273 | .104 | .717 |
| Gender=Female | 0^b^ | . | . | . | . | . | . | . | . |
| age | .002 | .0190 | .124 | .902 | -.035 | .040 | 1.002 | .965 | 1.041 |
| FSIQ | .020 | .0141 | 1.427 | .157 | -.008 | .048 | 1.020 | .992 | 1.049 |
| Probability distribution: Binomial  Link function: Logit^a^ | | | | | | | | | |
| a. Target: anymood | | | | | | | | | |
| b. This coefficient is set to zero because it is redundant. | | | | | | | | | |
