# Supplementary material for: Psychopathology in adults with copy number variants
Source: Psychol Med. 2022 Feb 11;53(7):3142–9. doi: 10.1017/S0033291721005201 (PMC10244007; doi:10.1017/S0033291721005201)
Supplement: Supplementary file 1 [file S0033291721005201sup001.zip › S0033291721005201sup005.docx]

Table S4:

When we removed the 33 participants with 22q11.2 deletion from the analysis we retained 25 probands and 66 non-probands. The odds ratios for the group comparisons of diagnoses were as follows:

| Category | Odds Ratio (in favour of proband group) | P | 95% CI of OR |
| --- | --- | --- | --- |
| Any psychiatric | 0.35 | 0.45 | 0.22-5.512 |
| NDD | 3.00 | 0.24 | 0.48-18.74 |
| Any psychotic | 7.77 | 0.19 | 0.37-164.88 |
| Anxiety | 0.57 | 0.53 | 0.10-3.31 |
| Mood disorder | 2.53 | 0.34 | 0.38-17.10 |

Group differences on GAF and IQ remained significantly different (VIQ: p-0.02; all other p’s < 0.001).
