# Supplementary material for: Psychopathology in adults with copy number variants
Source: Psychol Med. 2022 Feb 11;53(7):3142–9. doi: 10.1017/S0033291721005201 (PMC10244007; doi:10.1017/S0033291721005201)
Supplement: Supplementary file 1 [file S0033291721005201sup001.zip › S0033291721005201sup006.docx]

**Table S5: Physical phenotypes of all CNV carriers; N=124**

| Disease category | Specific disease/ syndrome | | | |
| --- | --- | --- | --- | --- |
|  |  | 22q11.2 DS  N=33 | Other CNVs  N=91 | Whole sample  N=124 |
| Cardiovascular disorders (CV)  N=40 [32.3%];  10 with 2 CV disorders | VSD  ASD  Tetralogy of Fallot  Mitral valve insufficiency  Congenital  Acquired  Aortic valve deformity  Aortic arch abnormality  Truncus arteriosus  Pulmonary stenosis  Hypertension  Hypotension  Ischaemic Heart Disease  Arrhythmia  Bundle branch block  Heart failure  WPW  Stroke  Raynaud’s  Giant Cell Arteritis | 7  1  3  1  1  0  0  1  1  1  3  3  0  1  0  0  0  1  1  1 | 0  0  0  2  1  1  1  0  0  0  8  2  4  2  1  1  1  2  1  0 | 7  1  3  3  2  1  1  1  1  1  11  5  4  3  1  1  1  3  2  1 |
| Gastrointestinal disorders (GI)  N=21 [16.9%]; 4 with 2 GI disorders | Congenital oesophageal stricture  Congenital bowel stricture  Congenital band gallbladder  Imperforate anus  Congenital gastric malformation  Meckel’s diverticulum  Gastric ulcer  Coeliac disease  Appendicitis  Fat liver  Cholecystitis/ gallstones  Inflammatory Bowel Disease  Polyps of gallbladder | 0  0  0  1  0  0  1  2  1  0  0  1  0 | 1  1  1  0  1  1  2  0  3  2  8  1  1 | 1  1  1  1  1  1  3  2  4  2  8  2  1 |
| Genitourinary disorders (GU)٭  N=13 [10.5%]  5 with 2 GU disorders | Renal agenesis (1 kidney)  Bicornuate uterus  Bladder/ ureter malformations  Hypospadias  Ovarian borderline tumours  Nephritis  Kidney stones  Interstitial cystitis  Renal failure  Bladder prolapse  Uterine prolapse | 2  0  1  0  0  0  0  0  0  0  0 | 0  1  0  1  2  4  2  1  1  1  1 | 2  1  1  1  2  4  2  1  1  1  0 |
| Respiratory disorders  N=32 [25.8%]; 2 with 2 respiratory disorders | Congenital subglottic stenosis  Congenital tracheal cyst  Asthma  COPD  Spontaneous pneumothorax | 1  0  9  1  1 | 0  1  21  0  0 | 1  1  30  1  1 |
| Endocrine disorders  N=34 [27.4%]; 1 with 2 endocrine disorders | Diabetes  Type I  Type II  Gestational  Type unknown  Hypothyroidism  Hyperthyroidism  Hypoparathyroidism  Thymus aplasia  Cryptorchidism  Polycystic Ovary Syndrome | 2  0  1  0  1  2  1  2  1  0  0 | 12  1  9 #  2  0  10 ##  0  0  0  1  4 | 14  1  10  2  1  12  1  2  1  1  4 |
| Orofacial syndrome  N=22 [17.7%] | Cleft palate  Overcrowding/ misaligned teeth  other | 7  1  3 | 1  2  8 | 8  3  11 |
| Musculo-skeletal (MS)  N=65 [52.4%]; 15 have 2 MS disorders, 9 have 3, 4 have 4 | Spinal deformities٭٭  Spina bifida  Brachial cleft sinus  Scapula alata  Hypermobile joints  Hernia٭٭٭  Hiatus  Inguinal  Unknown type  Congenital cubitus varus  Congenital pes planus  Congenital short Achilles tendons  Finger or toe malformations  Pilonidal abscess  Rheumatoid arthritis  Osteoarthritis  Arthritis unspecified  Polymyalgia rheumatica  Spondylitis  Meralgia paraesthetica  Crumbling teeth  Retained milk teeth  Chondromalacia patellae  Osteoporosis  Gout  Microcephalus | 9  1  0  0  1  6  1  2  3  1  1  1  4  0  1  0  2  1  0  0  4  1  1  3  0  0 | 7  0  1  1  9  3  2  0  1  0  2  0  2  3  3  7  4  0  1  1  2  1  0  0  1  1 | 16  1  1  1  10  9  3  2  4  1  3  1  6  3  4  7  6  1  1  1  6  2  0  0  1  1 |
| Immunological  N=64 [51.6%] | Recurrent infections | 25 | 39 | 64 |
| Sleep  N=6 [4.8%] | Obstructive Sleep Apnoea  Central Sleep Apnoea  Slow Wave Arousal Disorder  Unspecified sleep disorder | 0  0  1  1 | 3  1  0  0 | 3  1  1  1 |
| Hearing  N=23 [18.5%] | Impaired hearing  Of these: Hearing aids | 14  8 | 9  3 | 23  11 |
| Vision٭٭٭٭  N=7 [5.6%] | Papilloedema  Bilateral cataracts  Vertical nystagmus  Iritis  Optic ataxia | 2  1  0  0  0 | 1  0  1  1  1 | 3  1  1  1  1 |
| Neurological |  |  |  |  |
| Seizures  N=31 [25%] | Epilepsy  Febrile seizures | 2  3 | 22  4 | 24  7 |
| Other neurological disorders  N=24 [19.4%]; 1 has 3 disorders | Migraines  Hemiplegic migraine  Myoclonus  Dystonia  Intention tremor  Other tremor  Benign intracranial hypertension  Intracranial cyst  Tourette’s  Meningitis  Cerebral abscess  Subcortical frontal calcification | 2  0  2  1  0  1  0  0  0  2  0  1 | 8  1  0  0  2  2  1  1  1  0  1  0 | 10  1  2  1  2  3  1  1  1  2  1  1 |
| Dermatological  N=20 [16.1%]; 4 have 2 disorders | Eczema  Psoriasis  Alopecia  Disintegrating nails  Severe acne  Geographical tongue  Basal cell carcinoma  Hypersalivation  Calcium deposits skin  Iron deposits skin | 1  1  0  0  1  1  0  0  0  0 | 4  2  2  2  6  0  1  1  1  1 | 5  3  2  2  7  1  1  1  1  1 |
| ENT  N=25 [20.2%] | ‘Glue ear’/ grommets  Peritonsillar abscess  Tonsillectomy | 13  0  2 | 11  1  17 | 24  1  18 |
| Haematological  N=15 [12.1%] | Thrombocytopaenia  Hypocalcaemia  Hypercalcaemia  Hypokalaemia  Haemochromatosis  Neutropenia  Non-Hodgkin follicular lymphoma  DVT | 1  6  0  1  0  0  0  0 | 1  1  1  0  1  1  1  1 | 2  7  1  1  1  1  1  1 |

٭ Incontinence not considered, which was mainly reported for individuals with ID

٭٭mainly scoliosis

٭٭٭3 x hiatus (22q del, 1q21 del, 1q21 dup); 2 x inguinal (both 22q del); 1 hernia following lipoma op (22q del)/ 3 x unknown type (all requiring operations in childhood; 2 x 22q del, 1 1q21 dup)

٭٭٭٭Nearly all have corrected vision; majority had congenital squints

# non-22q Type II diabetes: 2 x 16p11.2 del; 2 x 1q21 dup; 3 x 17q12 dup; 2 x 15q13.3 del

## non-22q hypothyroidism cases: 3 x 1q21 del; 1 x 1q21 (TAR) dup; 1 x 15q11.2q12 dup; 4 x 15q13.3 del; 1 x 15q13.2-3 dup
